# Review of Online Evidence-based Practice Point-of-Care Information Summary Providers: Response by the Publisher of DynaMed

**DOI:** 10.2196/jmir.1622

**Published:** 2010-09-09

**Authors:** Brian S Alper

**Affiliations:** ^1^DynaMedEBSCO PublishingIpswich, MAUnited States

**Keywords:** Medical Informatics, Evidence-based medicine, Point-of-care

## Abstract

In response to Banzi's et al review of online evidence-based practice point-of-care resources published in the Journal of Medical Internet Research, the publisher of DynaMed clarifies his evidence-based methodology.

We congratulate Banzi and colleagues for publishing a review of online evidence-based practice point-of-care resources [1]. It is important that these resources are evaluated and summarized by evidence-based medicine experts who are not involved in the products being reviewed.

The adherence to evidence-based methodology for development and maintenance of these products is important for maintaining trust and validity in the content used by practicing clinicians. Banzi et al evaluated evidence-based methodology for point-of-care resources based on published marketing materials and editorial policies as stated on the websites of the publishers.

We were surprised to see a score for DynaMed (the product I am responsible for as editor-in-chief and medical director of EBSCO Publishing) which was less than 100% for our “evidence-based methodology”. In fact, “evidence-based methodology” is the fundamental methodology for how our editors are trained and how we work on a daily basis. A 7-step evidence-based methodology is the core process for updating and maintaining DynaMed [2].

The specific rating difference appears to be for the question “Are systematic reviews preferred over other types of publication?”. We should clarify that we absolutely prioritize systematic reviews (preferably Cochrane reviews) in our hierarchical approach to content consideration for inclusion, placement of content, and sources for deriving overall conclusions for evidence synthesis and overview statements. Individual study summaries are often deleted when included in a subsequent systematic review unless the individual studies offer additional information.

However, our editorial policy page referred to the concept without explicitly describing the hierarchy used. We have now updated our editorial policy page to make this more explicit [2]. This is not a change in editorial policy. We have been doing it for years, but in reaction to the Banzi review we have now improved the explicitness and transparency of our method.

Thank you to Banzi et al for bringing this to our attention.
